# Selective modulation of cortical population dynamics during neuroprosthetic skill learning

**DOI:** 10.1038/s41598-022-20218-3

**Published:** 2022-09-24

**Authors:** Ellen L. Zippi, Albert K. You, Karunesh Ganguly, Jose M. Carmena

**Affiliations:** 1 Helen Wills Neuroscience Institute, University of California Berkeley, Berkeley, CA 94720 USA; 2 Department of Electrical Engineering and Computer Sciences, University of California Berkeley, Berkeley, CA 94720 USA; 3 Neurology and Rehabilitation Service, San Francisco VA Medical Center, San Francisco, CA 94121 USA; 4 Department of Neurology, University of California, San Francisco, CA 94143 USA

**Keywords:** Neural decoding, Brain-machine interface

## Abstract

Brain-machine interfaces (BMIs) provide a framework for studying how cortical population dynamics evolve over learning in a task in which the mapping between neural activity and behavior is precisely defined. Learning to control a BMI is associated with the emergence of coordinated neural dynamics in populations of neurons whose activity serves as direct input to the BMI decoder (direct subpopulation). While previous work shows differential modification of firing rate modulation in this population relative to a population whose activity was not directly input to the BMI decoder (indirect subpopulation), little is known about how learning-related changes in cortical population dynamics within these groups compare.To investigate this, we monitored both direct and indirect subpopulations as two macaque monkeys learned to control a BMI. We found that while the combined population increased coordinated neural dynamics, this increase in coordination was primarily driven by changes in the direct subpopulation. These findings suggest that motor cortex refines cortical dynamics by increasing neural variance throughout the entire population during learning, with a more pronounced coordination of firing activity in subpopulations that are causally linked to behavior.

## Introduction

Learned behaviors are reinforced through mechanisms involving both cortical and subcortical structures^1–4^. Just as behavioral actions are reinforced, so is the cortical population activity required to efficiently produce these actions^5–7^. Studying mechanisms of cortical reinforcement underlying behavioral reinforcement can be challenging as the exact neural population controlling the desired behavior is unknown. Early studies of biofeedback demonstrated that activity in motor cortex can be reinforced and volitionally controlled using reward and sensory feedback of the firing rate^8,9^. Later, initial research on brain-machine interfaces (BMIs) showed that subjects could learn to control external devices (e.g. computer cursors or robotic arms) by learning to modulate the activity of a population of neurons and that the neural encoding of these prosthetic movements changed over time and decreased in variability ^10–14^. These BMIs allow for precisely defined mappings between recorded neural activity and behavior^10,13,15^. Studies leveraging BMIs to study learning-related changes in cortical activity have demonstrated that neuroprosthetic skill learning can require the production of novel cortical dynamics to obtain skillful control ^11,16,17^.

While classical approaches examine individual neurons to understand fundamentally how motor cortical activity is reinforced, more recent methods looking at population-level activity have uncovered how dynamic processes may govern movement planning and execution^18–29^ as well as learning^28,30–38^. Population-level activity is often characterized by low-dimensional dynamics that capture patterns of co-activation across neurons within a population^39^. These population-level dynamics arise from input connectivity and within-population connectivity. Two parallel mechanisms have been proposed to reinforce specific cortical population dynamics; fast reinforcement of dynamics that naturally produce a desired behavior and slower reinforcement that refines them to result in more reliable production of neural activity patterns^40,41^.

Previous studies have shown that neural populations are constrained to generate activity patterns within a pre-existing covariance structure within short timescales^30,32,37,38^, suggesting that it is faster to learn to control and repurpose pre-existing cortical population dynamics than it is to modify them. When decoder perturbations that change the behavioral output associated with specific neural activity were introduced after subjects had already achieved proficient control using a BMI there was an immediate deficit in performance. However, over training, subjects were able to recover performance of cursor control and furthermore, experienced a washout when the perturbation was removed, but only when the perturbation did not require alteration of the natural covariance pattern among the recorded neurons^32^. Other work has demonstrated that animals can learn to control BMIs that require neural patterns outside of the pre-existing covariance structure over the course of multiple days^5,31^. This eventual modification of cortical dynamics suggests that learning novel skills requires the production of new underlying population activity that develops over longer timescales.

With motor cortical BMIs, a small subset of all possible neurons in motor cortex is selected to use as input to the decoder (direct neurons). These neurons exist within a large network of other motor cortical neurons (indirect neurons). While the selection of direct neurons from all recorded units in our experiments was arbitrary and there was initially no functional difference between the those selected to be direct and those that are not, previous work has shown that differences in the neural activity of these two groups emerge with learning^16,42–47^. For example, it has been demonstrated that the task-relevant modulation of indirect neurons gradually reduces relative to direct neurons over learning^16^. Additionally, it has been shown in rodents that coherence develops between dorsal striatum and direct neurons, but not indirect neurons^44–46^. In a study using 2-photon calcium imaging to record neural activity, mice initially modulated activity of both direct and indirect neurons, but predominantly modulated direct activity after learning^42^. Thus, it is likely that the initial cortical dynamics that produce desirable outcomes involve both direct neurons and the surrounding cortical network. Over time, as these cortical dynamics are refined, they may adapt to exclude neurons that do not directly drive behavior.

If this hypothesis is true, we expect differences in how cortical population-level dynamics within direct and indirect subpopulations change over time as well. As cortical dynamics are modified for more efficient control, the direct subpopulation would be expected to undergo further modification than the indirect subpopulation as additional modifications to indirect activity would not directly result in desirable outcomes. Here, we investigate this idea by studying recorded ensembles of motor cortical neurons while only a subset was assigned to have a causal role during BMI control and characterize the differential changes in coordinated neural dynamics between direct and indirect subpopulations.

## Results

**Figure 1:**
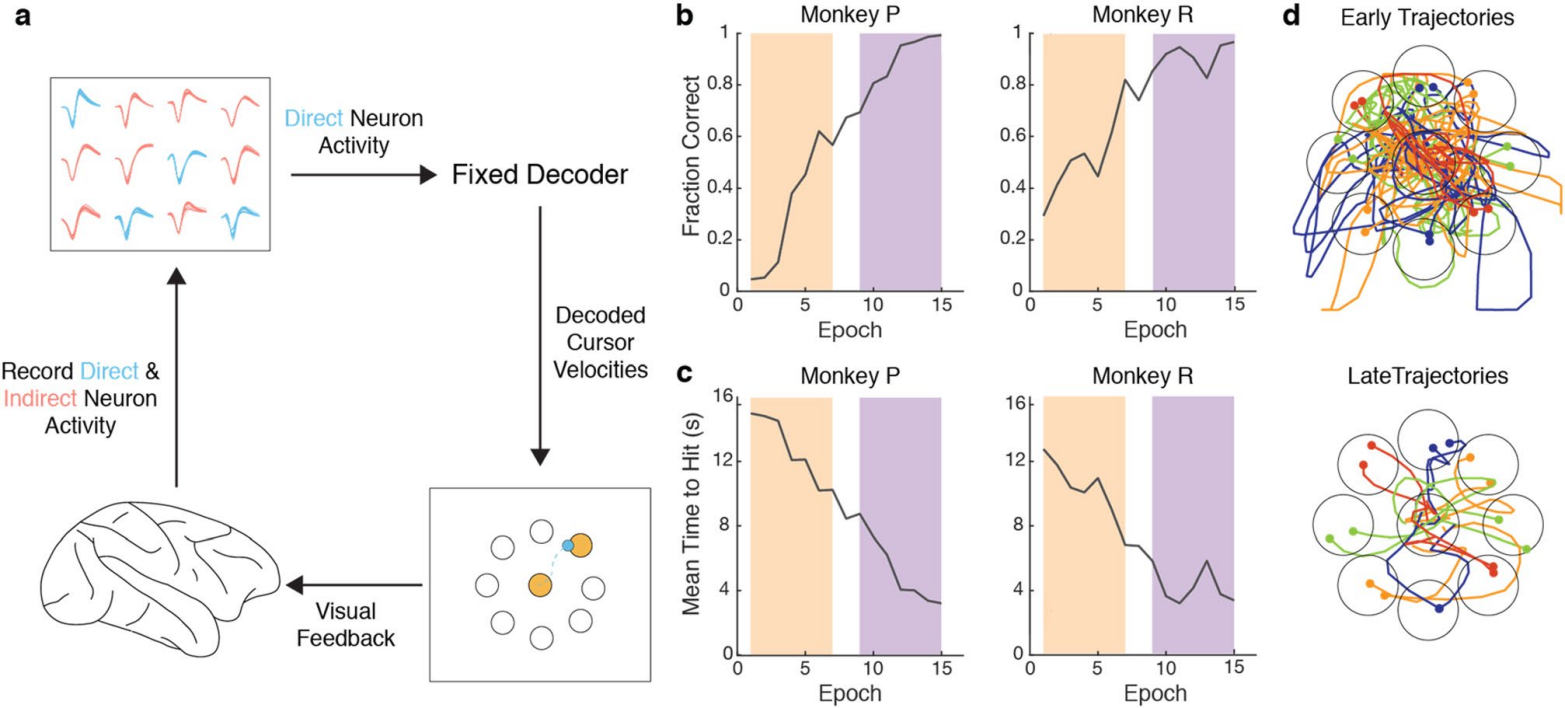
Experimental setup and behavioral performance. (**a**) Experimental setup from Ganguly and Carmena, 2009. Activity recorded from direct neurons (blue) in M1/PMd was input into a fixed linear decoder and used to drive a computer cursor to perform a center-out task (see Methods for details). Activity from indirect neurons (red) was simultaneously recorded, but was not input into the decoder. (**b**) Performance improves over the first 15 training epochs for Monkey P and Monkey R. Each training epoch consists of 150 initiated trials. For some analyses, epochs were divided into groups of early (orange) and late (purple). Fraction of initiated trials that were successful increased over training epochs. (**c**) The time to reach a target decreased over training epochs. (**d**) Representative examples of single-trial cursor trajectories during the early and late learning.

Two rhesus macaques (P and R) were chronically implanted with bilateral microelectrode arrays in primary motor and dorsal premotor cortices, with electrodes from a single hemisphere used for BMI control and subsequent analyses (see Methods). The monkeys learned to perform a two-dimensional, self-initiated, center-out BMI task, in which they drove a cursor under neural control to one of eight randomly instructed peripheral targets for a juice reward (Fig. 1a). The next trial was initiated by driving the cursor back to the center target. Trials from all days of the experiment were concatenated then separated into 150-trial epochs since the number of successful trials was lower in early days of learning. Both animals increased the fraction of successful trials (Fig. 1b) and decreased the time to move the cursor from the center target to the peripheral target (Fig. 1c) over the course of the first 15 epochs. Example cursor trajectories from early and late learning are shown in Fig. 1d. To capture the correlates of learning before performance saturated, only the first 15 epochs were used for each animal.

Because the BMI decoder used in the experiment was novel to the subjects, they had to initially explore the neural population activity space. Over time, the subjects learn from the behavioral consequences of explored activity patterns and select target-achieving, rewarded patterns of activity. To characterize the neural dynamics associated with this neuroprosthetic learning, we examined recorded ensembles of motor cortical neurons from which only a subset was assigned a causal role during control. We define these neurons, whose activity was used as a direct input to the BMI decoder, as “direct neurons” (Monkey P, N = 15; Monkey R, N = 10). The remaining recorded motor cortical neurons, recorded using the same two 4 × 4 mm 64-channel microelectrode arrays (interelectrode distance 500 um), whose activity was not used as direct input to the BMI, we define as “indirect neurons” (Monkey P, N = 29–69; Monkey R, N = 87–187). Spiking activity and waveforms for a representative direct and indirect unit are shown in Figure S1. For some analyses in which it is important to consider the same population across epochs we refer to indirect neurons that were stably recorded across all 15 epochs as “stable indirect neurons” (Monkey P, N = 17; Monkey R, N = 14). Stability of the indirect neurons was assessed using the methods described in Fraser and Schwartz^48^. This method uses pairwise cross-correlograms, the autocorrelogram, waveform shape, and mean firing rate to classify neurons and has previously been used on recordings obtained from chronically implanted microelectrode arrays to assess the stability of neurons across days^49–52^. Example waveforms from representative stable indirect units for each animal on the first day, middle day, and last day of recording are shown in Figure S2.

**Figure 2:**
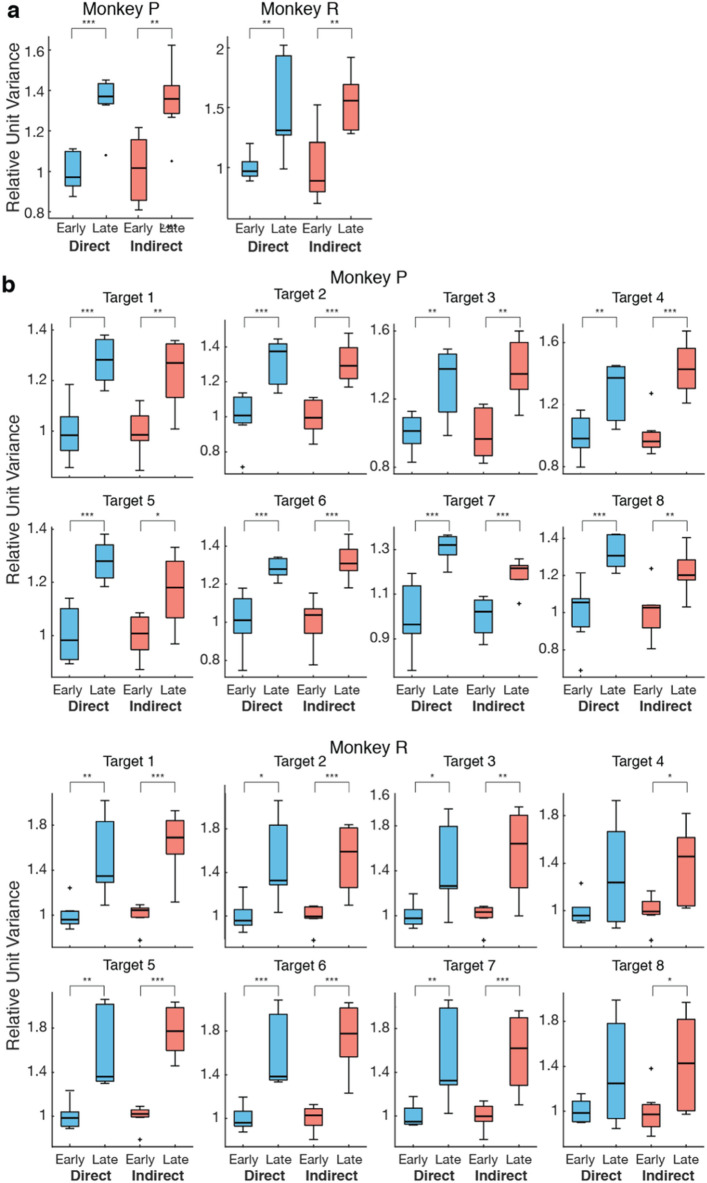
Neural variance increases with learning Variance was calculated for each neuron and then averaged across neurons. Relative variance was calculated by normalizing to the mean early variance within subpopulation. (**a**) Both direct and indirect subpopulations increased relative neural variance from early to late learning (Unpaired t-test; Monkey P: direct *p* = 8.63e-5, indirect *p* = 0.002; Monkey R: direct *p* = 0.007, indirect *p* = 0.003). (**b**) Analysis was repeated within target. Both direct and indirect subpopulations increased relative neural variance from early to late learning within target (Unpaired t-tests results reported in Table 1)

First, we examined how the neural firing rate variance changed in each subpopulation over learning. Changes in neural variance are often used as a proxy for neural exploration, as increasing the variance in firing rate allows for neurons to form different coordinated patterns of firing^5,36,53–56^. Past work has shown neural activity fires in more coordinated patterns as behavior stabilizes, thereby decreasing the dimensionality in neural space over learning^5,]^^30–32^. We commonly refer to these low-dimensional spaces as manifolds or neural subspaces. In order to observe changes in these neural subspaces, epochs were separated between early and late for each animal (Epochs 1–7 and Epochs 9–15, respectively) to track differences as behavioral performance improved. The firing rate for each neuron was binned in 100 ms intervals. The binned firing rate variance for each neuron was then averaged across all neurons for each epoch. In early learning, the mean firing rate variance was significantly higher in the direct subpopulation than the indirect subpopulation for one subject and there was no significant difference for the other (Fig. S3). We normalized the firing rate variance for each subpopulation based on the mean variance in early learning to assess relative change in variance from early to late learning within each subpopulation. In both animals, we observed an increase in relative unit variance between early and late learning for both direct and indirect subpopulations (Fig. 2a). This increase in variance suggests a concerted effort of neural exploration that exists in a broader network that includes both direct and indirect neurons. To ensure that this increase in variance was not due to a change in the distribution of time spent at each target, we repeated this analysis within each target (Fig. 2b). Trials to each target were evenly divided across 15 epochs and relative unit variance during early (first 7 epochs) and late learning (last 7 epochs) was considered for each target individually. The relative unit variance increased within both direct and indirect subpopulations for Monkey P and for six of the eight targets for Monkey R (Table 1), indicating that this result was not due to a change in the distribution of time spent at each target. Because the change in relative unit variance was consistent across targets, subsequent analyses included trials to all targets to increase the statistical power associated with more trials. Furthermore, because the task required two-dimensional control to achieve success at all targets, grouping trials across all targets provided better insight into how learning occurs in a generalized two-dimensional space rather than for target-specific activity.

**Table 1:** Relative variance within target. P-values from unpaired t-test comparing relative neural variance in early and late learning calculated within each target separately.

Target	Monkey P		Monkey R	
	Direct	Indirect	Direct	Indirect
1	1.74e-04	0.003	0.006	8.64e-05
2	9.01e-04	1.72e-04	0.010	7.72e-04
3	0.004	0.001	0.015	0.002
4	0.006	1.35e-04	0.117 (n.s.)	0.018
5	7.24e-05	0.012	0.001	2.12e-06
6	3.78e-04	1.25e-04	5.71e-04	4.51e-05
7	3.17e-04	5.25e-04	0.005	9.75e-04
8	6.07e-04	0.006	0.093 (n.s.)	0.037

Previous work has shown that neurons fire in increasingly coordinated patterns as performance improves^5^. We consider these changes in coordinated firing as a proxy for consolidation of neural population dynamics since the neural variance is stabilizing onto low-dimensional subspaces. We use factor analysis (FA) to separate the neural variance in the population into two components—private and shared variances^57^. The shared variance is the variance between neurons in the population and can be thought of as the underlying correlated firing pattern in the recorded population^19,58^. Conversely, the private variance denotes the amount of variance each neuron has that is independent from the rest of the population. Past studies have explored the roles of these components in the direct neuron population, showing private variance as a proxy for exploration while an increase in shared variance is correlated to skill consolidation^5,59^. Previous work has quantified the amount of coordinated neural activity as a measure of the balance between shared and private variance: the ratio of shared variance over total variance (SOT)^5,58–61^. Here, we compared the proportion of the total neural variance that is captured in shared spaces for the combined direct and indirect population in each epoch as an estimate of coordination within the recorded population across learning.

**Figure 3:**
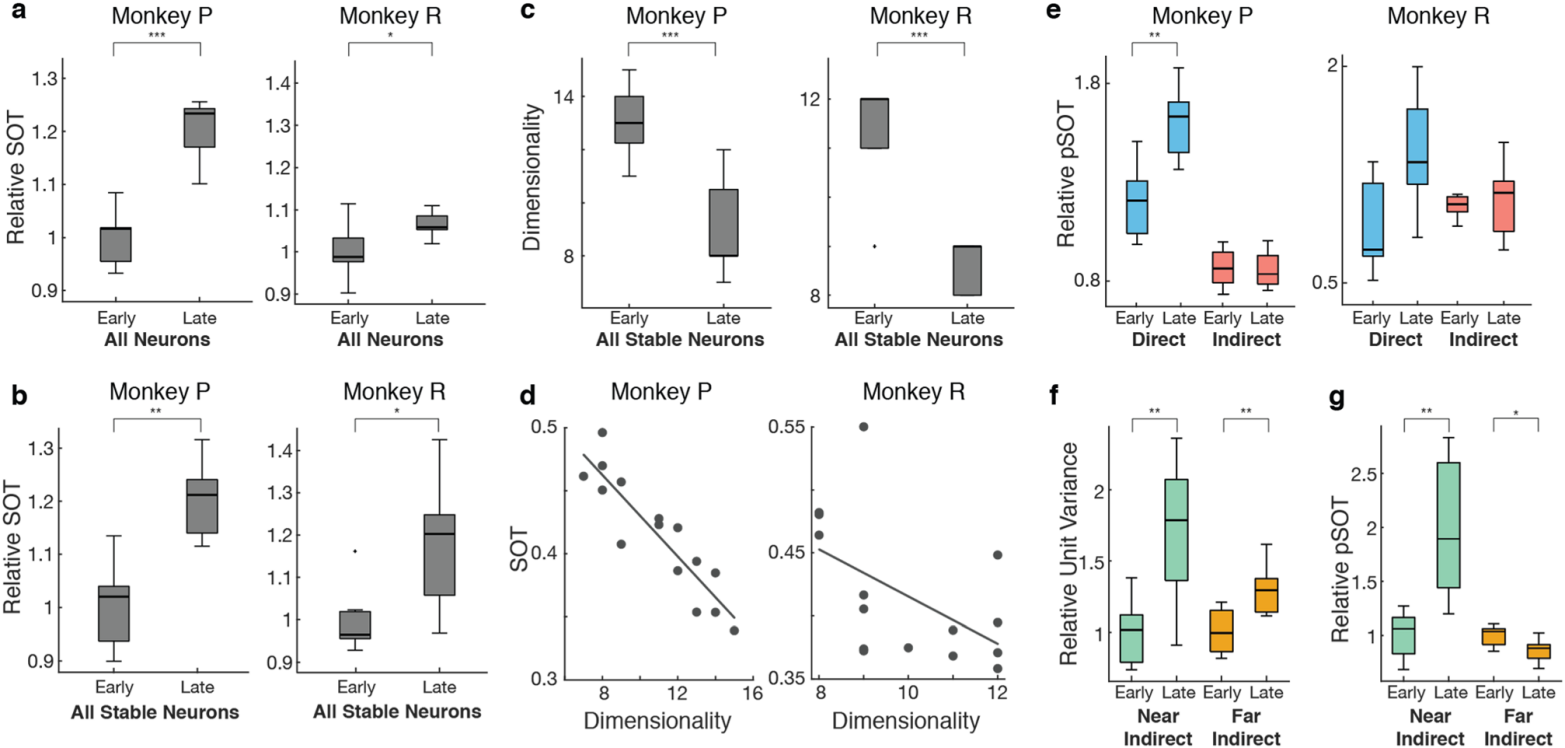
Increases in coordinated neural activity over learning are primarily driven by direct neurons. (**a**) The relative shared-over-total variance (SOT) ratio was calculated with respect to the mean early SOT across the entire recorded population. Relative SOT increased between early and late learning, indicating an overall increase in coordination of neural activity in the entire recorded population (Unpaired t-test; Monkey P, *p* = 1.31e-5; Monkey R, p = 0.033). (**b**) Relative SOT also increased between early and late learning for a stably recorded population consisting of the same units each epoch (Paired t-test; Monkey P, *p* = 0.002; Monkey R, *p* = 0.027). (**c**) Dimensionality of the neural subspace for the stably recorded population decreased from early to late learning (Unpaired t-test; Monkey P: p = 4.10e-4; Monkey R: p = 8.24e-5). (**d**) The dimensionality of the neural subspace is correlated with SOT (Linear regression; Monkey P: R^2^ = 0.813, *p* = 4.41e-6; Monkey R: R^2^ = 0.277, *p* = 0.044). (**e**) Respective contributions of each sub-population to the SOT ratio (pSOT, see Methods for details) were calculated in early and late learning relative to contributions in early learning. Only direct pSOT relative to early learning increased from early to late learning (Paired t-test; Monkey P, direct *p* = 0.003, indirect p = 0.805; Monkey R, direct p = 0.084, indirect p = 0.675). To test that the change in direct was not due to a chance grouping of neurons, we repeated the t-test 500 times while shuffling direct and indirect labels and compared the true direct t-statistic to a distribution of t-statistics from the shuffled populations (Permutation test; Monkey P, direct *p* = 0.014; Monkey R, direct *p* = 0.078). (**f**) Both near and far indirect neurons exhibited significant increases in neural variance (Unpaired t-test; near p = 0.006, far *p* = 0.007). (**g**) Only near indirect neurons exhibited a significant increase in pSOT relative to early learning (Unpaired t-test; *p* = 0.003). Far indirect neurons exhibited a significant decrease in pSOT (Unpaired t-test; *p* = 0.022). To test that changes in pSOT are not due to a chance grouping of neurons, we repeated the t-tests 500 times while shuffling near and far labels (Permutation test; near, *p* = 0.008; far, *p* = 0.002).

Since the indirect population consisted of different units each epoch, we normalized the SOT to the mean SOT for early epochs (Fig. 3a). In both animals, we found that the relative SOT increased between early and late learning for the entire recorded population including both direct and indirect neurons. Together, with the increase in variance over learning, our results indicated a high level of increased coordination that occurs within the entire recorded population driven by increased exploration as BMI performance improves. To assess that the effect was not due to day-to-day differences in the population, we conducted the same analysis on neurons that were stably recorded across all 15 epochs, which yielded consistent results (Fig. 3b). Along with an increase in SOT, previous work has shown learning-related decreases in dimensionality of the shared neural subspace for the direct subpopulation^5^. We found a similar decrease in dimensionality for the entire stably recorded population (Fig. 3c) and furthermore found that the dimensionality of the neural subspace in each epoch was significantly correlated with the SOT of each epoch (Fig. 3d).

While the increase in SOT indicates more coordination within the entire population, it does not explain whether these changes are driven by a specific subpopulation. To answer this question, we considered the partial shared-over-total variance (pSOT) ratio of the stably recorded population to see how the same population of neurons change coordinated firing activity over learning (see Methods). Intuitively, the pSOT ratio asks how much of the overall change in coordinated activity was driven by one subpopulation versus the other. We see that, relative to early learning, there was an increase in pSOT_direct_ in late learning but not in pSOT_indirect_ for both animals (Fig. 3e), indicating that the increase in coordination of population activity seen across the stably recorded population was driven by the increase in coordination of population activity within the direct subpopulation. While this result was only statistically significant in Monkey P, Monkey R (who had fewer direct neurons) exhibited the same trend. The larger increase in pSOT_direct_ suggests that the increased neural exploration in the network was primarily a consequence of changes in coordinated patterns specific to the direct neurons. To further characterize these changes in the indirect neurons, we separated all of the indirect neurons in Monkey P into “far” and “near” indirect neurons. “Far” indirect neurons (N = 29–69) were those recorded on electrodes not containing direct neurons. In contrast, “near” indirect neurons (N = 7–14) were indirect neurons that existed on the same electrode shanks as direct neurons. Monkey R was excluded from these analyses due to recording too few near indirect neurons during several epochs (N = 0–10). We found that neural variance increased for both far and near indirect neurons between early and late learning (Fig. 3f). However, the pSOT only increased for the near indirect subpopulation and significantly decreased for the far indirect subpopulation (Fig. 3g). Together, these results suggest that while neural exploration exists in broader networks consisting of both direct and indirect neurons, activity in neurons closer in proximity to direct neurons becomes more coordinated than activity in neurons farther away from direct neurons. While the probability of synaptic connections among neurons does not depend strongly on interneuron distance, differences in near and far indirect neurons could be due to common synaptic inputs between the direct neurons and near indirect neurons from other brain regions into M1^62^.

**Figure 4:**
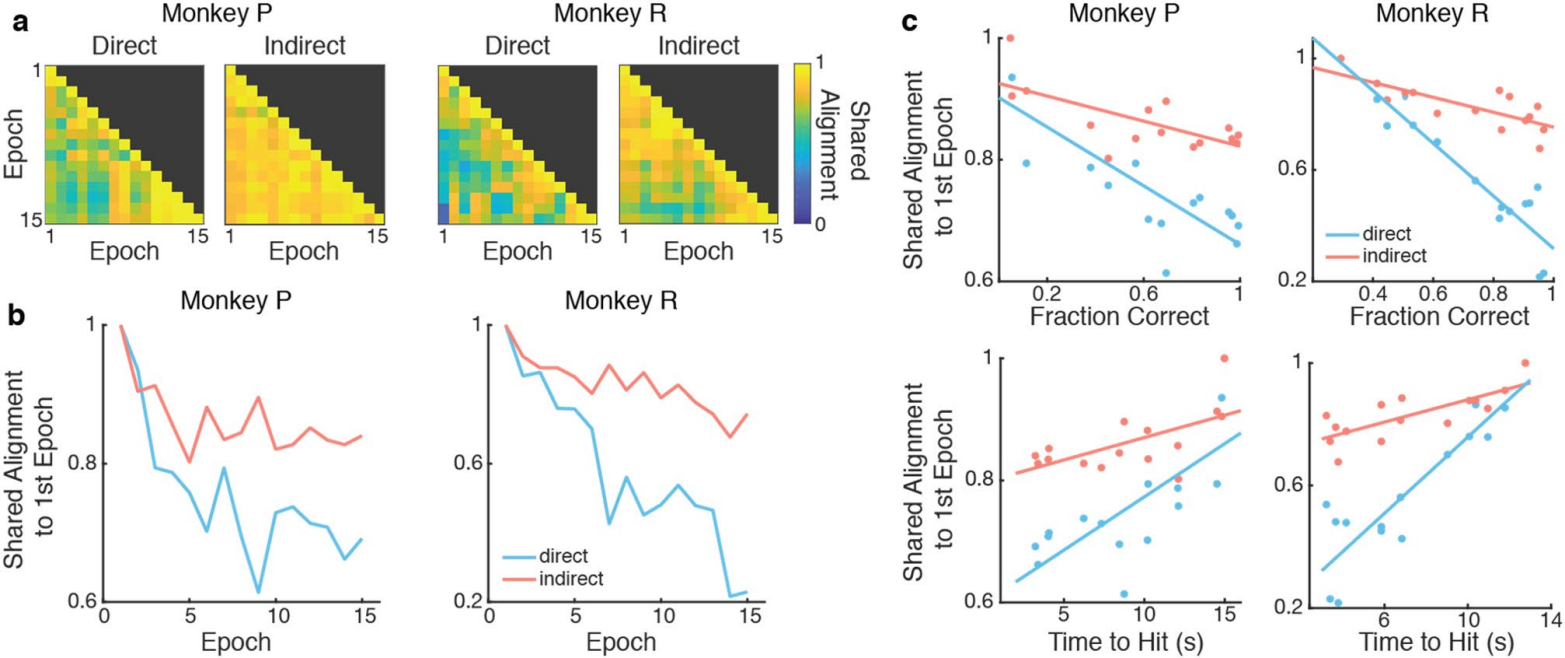
Rotation of low-dimensional neural subspace. (**a**) Shared alignment was calculated pairwise between epochs for each subpopulation. (**b**) Alignment diverges from the first epoch in both subpopulations (Linear regression; Monkey P: direct, R^2^ = 0.530, *p* = 0.001, indirect, R^2^ = 0.352, *p* = 0.012; Monkey R: direct R^2^ = 0.857, *p* = 4.63e-7, indirect R^2^ = 0.760, *p* = 1.41e-5). The slopes for direct and indirect shared alignment across epochs were significantly different (One-way ANCOVA; Monkey P: *p* = 0.024; Monkey R: *p* = 5.84e-6). (**c**) Alignment is correlated with fraction correct (Top, linear regression; Monkey P: direct, R^2^ = 0.657, p = 2.0e-4, indirect R^2^ = 0.481, *p* = 4.2e-4; Monkey R: direct R^2^ = 0.870, p < 1.0e-6, indirect R^2^ = 0.589, *p* = 8.0e-4) and time to hit (Bottom, linear regression; Monkey P: direct, R^2^ = 0.535, *p* = 0.002, indirect R^2^ = 0.380, p = 0.014; Monkey R: direct R^2^ = 0.815, *p* < 1.0e-6, indirect R^2^ = 0.592, *p* = 8.0e-4).

To characterize how neural exploration modified the direct and indirect neural subspaces differently, we quantified these changes by calculating the shared alignment pairwise between each training epoch’s shared covariance matrix for each subpopulation according to the methods described in Athalye et al., 2017 (Fig. 4a). The shared alignment measures the similarity of covariance planes to compare how much of the shared space of one epoch projects onto the shared space of another epoch. Intuitively, given both two-dimensional shared subspaces, the shared alignment compares the angle between the two planes. Orthogonal planes, or subspaces, would result in a shared alignment of 0 and perfectly aligned planes would result in a shared alignment of 1. If the shared subspace consolidates with learning, as has been shown in direct subpopulations^5^, we would expect the shared subspace to rotate away from the initial subspace over learning. If the shared subspace remains fixed over learning, we would predict that the alignment between the first epoch and later epochs remains high, indicating little change in the coordinated activity of the population. Since we are interested in how the subspaces pertaining to specific populations change over time, we analyzed only the neurons that were stable across learning. We found that the shared alignment decreased from the first epoch for both subpopulations (Fig. 4b). This indicates that both subpopulations rotated their low-dimensional subspaces, suggesting that neurons may adapt on a network level that includes both direct and indirect neurons. Furthermore, this rotation of the low-dimensional subspaces is correlated with behavior (Fig. 4c). As the shared subspace diverges from where it began in the first epoch, the fraction of correct trials significantly increases and the time it takes for the cursor to reach the target significantly decreases. While this is true for both the direct and indirect subpopulations, the extent of rotation as measured by the shared alignment with the first epoch and the proportion of the variation in the shared alignment that is predictable from the behavior were higher for the direct subpopulation than the indirect subpopulation.

**Figure 5:**
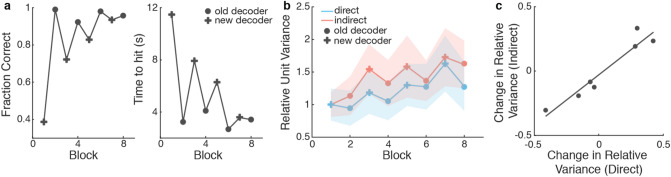
Neural variance modulates concomitantly between subpopulations. (**a**) Monkey P learned to perform BMI with a new decoder following proficient control with the old decoder. 8 experimental blocks were performed, alternating between a new decoder (cross) and the previously learned decoder (circle). Fraction of initiated trials that were successful increased over training blocks (left) and the time to reach a target decreased over training blocks (right). (**b**) Both subpopulations increase their neural variance over blocks (Linear regression; Direct R^2^ = 0.623, *p* = 0.020, Indirect R^2^ = 0.655, *p* = 0.015). The relative variances across blocks are correlated between subpopulations (Pearson’s r, r = 0.856, *p* = 0.007). (**c**) Each point represents the change in relative variance between two consecutive blocks. The changes in relative variance within the direct and indirect subpopulations are correlated (Pearson’s r, r = 0.820, *p* = 0.024).

To further explore how indirect and direct neural activity may adapt together, we analyzed data from a second experiment in which Monkey P learned to perform the same BMI task with a new decoder following proficient control with the original learned decoder (Fig. 5a). The new decoder used the same direct neurons as the original decoder but the decoder weight assigned to each direct neuron was changed so that the same activity patterns result in different cursor movements when using the different decoders. Eight experimental blocks were performed over the course of four days, alternating between control with the new and previously learned decoder each day. The neural variance for direct and indirect neurons was calculated within each of these eight blocks. Note that only stable indirect neurons were used for this analysis since we wanted to explicitly track how the variance changed as a function of block number. We found that both subpopulations increased and decreased neural variance together over blocks, with similar changes in variance between blocks occurring in both direct and indirect neurons (Fig. 5b). Thus, increases in neural variability with changing decoders over shorter timescales involved increased exploration not only by the direct neurons, but also by the supporting indirect neurons. Because there was an increase in neural variability in both subpopulations with each decoder swap, we were also able to assess whether the amount change in neural variance was similar between the two subpopulations. We found that the changes in the firing rate variance of the direct subpopulation were correlated to changes in the indirect subpopulation (Fig. 5c).

## Discussion

In this study, we explored how changes in cortical population dynamics underlie skill learning. Specifically, we examined how cortical dynamics of subpopulations of neurons change over learning when the mapping of neural activity to behavior is precisely defined using a BMI. That is, how does the adaptation of the subpopulations used as inputs into a BMI decoder (direct neurons) compare to that of a subpopulation not used for decoding (indirect neurons)?

Our results revealed that learning-related neural state space exploration included neurons from both the direct and indirect subpopulations. Both subpopulations increased variance in their firing rates (Fig. 2) and there was an increase in coordination of neural firing patterns across the combined population that was correlated with a decrease in dimensionality of the neural space (Fig. 3). Previous literature has suggested that volitional modulation of neural activity in animals may be related to movements, cognitive imagery, or shifts in attention^63,64^. Thus, it is possible that these learning-related changes in neural variance are the result of an underlying behavioral strategy through which the animals learn to modulate their neural activity. While we could not directly compare these changes in neural variance and coordinated activity to undetected movements made by the animals, both animals were observed to have minimal movements during BMI control^11^.

An increase in neural variability with learning, as observed in our results, has also been seen in previous studies^5,36^. One explanation for this increase in variability is that it allows the brain to explore new activity patterns that may improve behavior^5^. Another study finding the same increase in neural variability in early learning proposed that this increase may be the result of changing internal states or increased neural engagement^36^. Thus, this increase in variance of the firing rates of both direct and indirect neurons may be a result of an increased exploratory drive or a change in the animals’ internal state, which may be indicative of its arousal or uncertainty about its environment.

Previous work observing increases in coordinated population activity suggested that these changes are characteristic of more stereotyped behavior over learning and this increased neural covariance has been associated with subjects making straighter, more direct paths to the targets^5^. Furthermore, changes in covariance structure have been shown to relate to synaptic connectivity^65^. Thus, when considering the entire recorded population, it appears as though the whole population adapts together to facilitate learning. However, past work has shown differences in adaptation between direct and indirect neurons^16,43,45^. When we considered the relative contribution of each subpopulation to the overall increase in coordination, we found that the indirect subpopulation contributed very little to the increase in coordinated patterns (Fig. 3e). That is, while we witnessed an increase in coordination in the entire recorded population, there was less within-group coordination in indirect neurons compared to direct neurons. Our metrics of coordination (e.g., SOT) rely on averaging the amount of correlated activity between pairs of neurons. Coordination of firing activity occurring more heavily in one subpopulation would nevertheless increase the SOT in the entire population. Altogether, while both subpopulations exhibit similar levels of exploration over learning as demonstrated by an increase in neural variance, the exploration by the indirect subpopulation results in less of an increase in coordination of neural firing patterns than that of the direct subpopulation.

Changes in coordinated neural activity resulted in rotations of the neural space over learning in both direct and indirect subpopulations (Fig. 4). The neural space can be intuitively thought of as the lower-dimensional space in which co-activations of neurons exist and rotating this neural space corresponds to adjusting which neurons are more active given the state of other neurons in the population. Following this interpretation, both direct and indirect subpopulations similarly adapted their coordinated firing patterns within subpopulation over learning. This adaptation in coordinated firing patterns was also correlated with behavioral improvements in the task for both the direct and indirect subpopulations, however, the relationship was stronger for the direct subpopulation than the indirect subpopulation for both subjects. This result, along with the increase in pSOT observed in the direct subpopulation, suggests that as the direct subpopulation increases coordination of neural activity its covariance exhibits greater changes than that of the indirect subpopulation over learning.

We also examined how the neural firing rate variance of both the direct and indirect subpopulations changed in an experiment where two decoders were swapped each day, requiring changes in neural activity over short timescales. We found that changes in neural variance occurred with each decoder swap and were proportional between the two subpopulations (Fig. 5). This suggests that increases in neural variability occur over both short timescales, as seen in this experiment, and over longer timescales, as seen in the initial 15 epochs, involve both neurons within the supporting cortical network and direct neurons. Furthermore, the changes in neural variance between the two subpopulations were correlated. Consequently, both the direct and indirect subpopulations may be adapting via the same mechanism but to different extents. However, this experiment was limited to only four days of switching between decoders. It is unclear whether or not these parallel changes in neural variance between the direct and indirect subpopulations would continue if the animal was given more extensive practice.

Our findings that both direct and indirect subpopulations increase neural variability similarly but exhibit differential changes in coordination could be explained by existing hypotheses on how the brain learns to refine coordinated neural dynamics^40^. Specifically, small networks of cortical neurons may be driven by upstream cortical and subcortical inputs. We found that indirect neurons adapted in similar ways as direct neurons, suggesting that some indirect neurons may in fact be adapting with or alongside direct neurons. When we investigated how distance from direct neurons influenced these results, we found that indirect neurons in closer spatial proximity to direct neurons increased coordination more than indirect neurons that were farther away (Fig. 3g). While the upstream projections from cortical and subcortical structures are not necessarily spatially organized, our results are consistent with the hypothesis that upstream structures may be driving changes in smaller groups of inter-connected neurons^35^. Furthermore, it has previously been shown that when disparities are present between the control space and neural space (i.e. how well the decoders aligned to the natural firing patterns of the neurons), neurons with larger disparities adapt more over learning compared to neurons with smaller disparities^5,17,66,67^. A recent study has shown that task-related neurons, consisting of direct neurons as well as task-modulated indirect neurons, increase coherency to slow-wave activity (SWA) during sleep which has been linked with consolidation^43^. This suggests that in addition to online task practice, neural reactivations during sleep can aid in exploring the contributions of direct and indirect neural populations relative to successful outcomes and reward. Notably, indirect neurons that were closely tied to reward were preserved and resembled direct neurons; this might explain why some indirect neurons were modified during neuroprosthetic skill acquisition. This also provides further evidence that mechanisms of reinforcement learning may underlie our observed phenomena. Thus, it is quite plausible that adaptation of neural activity over BMI learning is attributed to finding the clusters of neurons with a direct effect on behavior, which may include both direct and indirect neurons, depending on their specific network connectivity and temporal association with successful outcomes.

In this study, we used factor analysis (FA) to find correlations in the neural activity of the recorded population. Underlying this model are latent factors, which are variables that coarsely group neurons together based on coordinated activity patterns. Importantly, the activity of a single neuron can be associated with multiple latent factors. While we were agnostic to what the latent factors in FA may correspond to in this study, they may be physiologically analogous to upstream connections from cortical or subcortical structures that drive changes in small clusters of neurons containing direct neurons. The idea that neural reinforcement is dependent on cortico-cortical and cortico-striatal circuits, similar to behavioral reinforcement, has been previously supported by studies using BMIs. For example, as rodents learned to produce specific patterns of cortical activity, coherence between these neurons and dorsal striatum emerged and neurons in dorsal striatum developed target-predictive modulation of firing activity^44–46^. Furthermore, mice without functional NMDA receptors in striatal projection neurons could not learn to re-enter a cortical pattern that led to reward. Thus, cortico-striatal plasticity is necessary for learning to efficiently produce the cortical activity patterns required to obtain rewards. These findings along with the results from our study further support the hypothesis that smaller clusters of neurons, which may include both direct and indirect neurons, are adapted over learning more than clusters of neurons that do not drive behavior.

Overall, our results demonstrate that the brain learns to modify cortical population dynamics in subpopulations relevant for behavioral control. When using a BMI, we find that neurons with direct input to the decoder as well as neurons in the surrounding cortical network increase exploration and consolidate their firing activity onto a low-dimensional neural space. The degree of coordination among the population is dependent on the relationship of the neural activity to the behavioral output. Thus, the brain may not be reinforcing the activity of single neurons, but rather reinforcing cortical population dynamics that are relevant to producing a desired behavior. These findings indicate that the brain learns to control a BMI by refining cortical population-level dynamics, suggesting that BMI decoders extracting information based on population-level statistics, such as the covariance structure of the population, may be more effective compared to traditional decoding methods based on the statistics of individual neurons. Dimensionality reduction techniques such as FA allow us to pull out the correlated activity in a population of neurons. That is, the shared variance obtained using FA represents the concerted activity of a population and if we assume that the uncorrelated activity is largely noise, then using these population-level statistics effectively increases the signal-to-noise ratio of the neural activity. Building decoders based on these smoothed neural signals may translate to smoother output signals (e.g. cursor movements).

In conclusion, this study demonstrates an emergence of coordinated population dynamics within both populations of neurons whose activity is directly used as input for BMI control as well as within the surrounding network. The extent to which these subpopulations modify their coordinated activity varies, with the direct subpopulation exhibiting larger changes. Understanding the role of modifications of adjacent indirect activity in obtaining precise control of a BMI may help us understand the neural adaptation that is required for achieving long-term, stable control of a BMI.

## Methods:

### Experimental design

#### Animal subjects

All procedures were conducted in compliance with the NIH Guide for the Care and Use of Laboratory Animals and were approved by the University of California at Berkeley Institutional Animal Care and Use Committee.

Two adult male rhesus monkeys (Macaca mulatta) were chronically implanted in the brain with arrays of 64 microelectrodes (Innovative Neurophysiology, Durham NC)^11^. Monkey P was implanted in the left hemisphere in the arm area of both primary motor cortex (M1) and dorsal premotor cortex (PMd), and in the right hemisphere in the arm area of M1, with a total of 192 microwires across three implants. Monkey R was implanted bilaterally in the arm area of M1 and PMd (256 microwires across four implants). Only activity from M1 was included in the direct ensembles (Monkey P: right M1; Monkey R: left M1) and only activity from the same hemisphere was included in the indirect ensemble. Array implants were targeted for pyramidal tract neurons in layer 5. Localization of target areas was performed using stereotactic coordinates from a neuroanatomical atlas of the rhesus brain^68^.

#### Electrophysiology

Neural activity was recorded using the MAP system (Plexon, Dallas TX). Stable units, to be part of the direct ensemble, were selected based on waveform shape, amplitude, relationship to other units on the same channel, interspike interval distribution, and the presence of an absolute refractory period. Only units from primary motor cortex were used which had a clearly identified waveform with signal-to-noise ratio of at least 4:1. Activity was sorted prior to recording sessions using an online spike-sorting application (Sort Client; Plexon). Stability of waveforms was confirmed by analyzing the stability of PCA projections over days (Wavetracker; Plexon).

Direct units are defined as the units being used to control the BMI. Indirect units consisted of the remaining recorded units. For analyses including only stable units from the same hemisphere, stability in the indirect ensemble was assessed using pairwise cross-correlograms, autocorrelograms, waveform shapes, and mean firing rates^48^.

#### Experimental setup and behavioral training

##### Manual control training before BMI

Before starting the BMI learning experiments, subjects were overtrained on the task performed with arm movements using a Kinarm (BKIN Technologies, Kingston ON) exoskeleton which restricted shoulder and elbow movements to the horizontal plane.

##### BMI tasks

Data from Ganguly and Carmena^11^, in which subjects performed a self-initiated, eight-target, center-out reaching task, was analyzed. In these experiments, a cursor on a screen was continuously controlled by neural activity. Subjects self-initiated trials by moving the cursor to a center target. One of the eight peripheral targets was randomly selected each trial. Self-initiated trials consisted of those in which the animal moved the cursor to the center target and held for 250-300 ms. Successful trials required the animal to move the cursor to the peripheral target within 15 s of initiating the trial and hold the cursor at the target for 250-300 ms. Successful trials resulted in a juice reward; failed trials were repeated. During BMI control, both arms were lightly restrained to restrict arm movement during BMI control. During selected sessions, video and surface electromyogram (EMG) recordings from proximal muscle groups were performed to confirm minimal arm movements occurred during BMI control.

After the initial 19-days of performing the BMI task with a fixed-decoder, Monkey P learned a second decoder over the course of four days. Within each of these four days, Monkey P performed one training block of the new decoder, followed by one training block of the old decoder. Both of these decoders were fixed and used the same direct ensemble as input.

#### Preprocessing pipeline

For all analyses, neural data was binned into 100 ms bins to match the decoder timescale. Additionally, learning was analyzed over “training epochs,” where each epoch consisted of 150 self-initiated trials. Learning took place over the first 15 training epochs (2250 self-initiated trials). We chose to analyze the data across training epochs, rather than days, to eliminate the effect of variable numbers of trials each day. Only the first 15 training epochs (2250 self-initiated trials) were analyzed; we defined early and late learning as the first and last seven of these training epochs, respectively. Monkey P initiated a total of 3589 trials. Monkey R initiated a total of 2357 trials.

### Statistical analysis

#### Factor analysis

##### Shared-over-total variance ratio

Factor analysis (FA) was conducted on the neural population for each epoch to observe underlying correlated neural activity. FA decomposes population signals into correlated and uncorrelated components. For a given neuron i, correlated activity is represented by the shared variance ${\Sigma }_{ii}^{shared}$, and the degree to which the activity was correlated over learning was represented by the ratio of shared-over-total variance of the neural population (SOT). We calculated the SOT ratio according to the methods described in Althaye et al^5^.

$$SOT=\frac{trace\left({\Sigma }^{shared}\right)}{trace\left({\Sigma }^{total}\right)}$$

Since FA decomposes the neural activity into correlated and uncorrelated components, we can reduce the dimensionality of the neural data by examining how much of the neural variance is captured by the shared (correlated) components. The number of dimensions was selected such that 90% (or more) of the shared variance was captured. A scree plot quantifying the variance captured by each factor for the combined population is shown in Figure S4.

##### Total variance

We also considered how the total variance changed between early and late learning. This was the sum of the private and shared variances ${\Sigma }_{ii}^{total}={\Sigma }_{ii}^{shared}+{\Sigma }_{ii}^{private})$.

##### Partial shared-over-total variance ratio

We quantified the respective contributions of subpopulations to the SOT ratio using the partial shared-over-total variance (pSOT) ratio. Here, we compared the sum of the shared variance for each subpopulation over the total variance for the entire population. A relative measure was used to account for the fact that the direct and indirect ensembles were different sizes. That is,

$$pSO{T}_{direct}=\frac{trace\left({\Sigma }_{direct}^{shared}\right)}{trace\left({\Sigma }_{all}^{private}\right)+ trace\left({\Sigma }_{all}^{shared}\right)}$$

$${pSOT}_{indirect}=\frac{trace\left({\Sigma }_{indirect}^{shared}\right)}{trace\left({\Sigma }_{all}^{private}\right)+ trace\left({\Sigma }_{all}^{shared}\right)},$$

where i denotes the covariance matrix for the (sub)population i.

##### Shared space alignment

We used the ‘‘shared space alignment’’ to measure the similarity between the shared variance of two different training epochs. The shared space alignment is the fraction of shared variance from one epoch captured in the shared space of a second epoch and thus ranges from 0 to 1. We calculated shared alignment according to the methods described in Athalye et al.^5^. Given two epochs, A and B, we first compute the projection matrix into Epoch B’s shared space, $col\left({U}^{B}\right)$.. We then project ${\Sigma }^{A,shared}$ onto B’s shared space, ${P}_{{U}^{B}}{\Sigma }^{A,shared}{P}_{{U}^{B}}^{T}$. Finally, the alignment is calculated,

$$Shared\, Alignment=\frac{trace\left({P}_{{U}^{B}}{\Sigma }^{A,shared}{P}_{{U}^{B}}^{T}\right)}{trace\left({\Sigma }^{A,shared}\right)}$$

##### Quantification and statistical analyses

All analyses were performed on each training epoch separately. Trends were analyzed for significance with linear regressions. Epochs 1–7 and Epochs 9–15 were grouped into “early” and “late,” respectively. Epoch 8 was omitted so that there were equal numbers of epochs for both early and late learning. Groupings of early and late epochs were compared using an unpaired two-sample t-test when comparing unstable populations of neurons and a paired one-sample t-test when comparing stable populations. Significance was considered as *p* < 0.05. A single asterisk was used to denote significance of at least *p* < 0.05, a double asterisk was used to denote *p* < 0.01, and a triple asterisk was used to denote *p* < 0.001.

For t-tests resulting in a significant change in one group but not the other (e.g. direct but not indirect; near but not far), a random permutation test in which the group labels were shuffled 500 times was calculated to ensure that the results were not a consequence of a small sample size. In all cases, the t-statistic for the group with the significant change was outside 95% of the distribution of t-statistics obtained from the shuffled labels tests indicating that the significant group was not a chance combination of neurons.

### Ethical approval

All procedures were conducted in compliance with the NIH Guide for the Care and Use of Laboratory Animals and reporting in the manuscript follows the recommendations in the ARRIVE guidelines. All procedures were approved by the University of California at Berkeley Institutional Animal Care and Use Committee.

## Supplementary Information

Supplementary Information.

## Data Availability

The datasets analyzed during the current study will be publicly available on Figshare  (https://figshare.com/articles/dataset/Selective_modulation_of_cortical_population_dynamics_during_neuroprosthetic_skill_learning/21111688)  upon publication.
