# Supplementary material for: Selective modulation of cortical population dynamics during neuroprosthetic skill learning
Source: Sci Rep. 2022 Sep 24;12:15948. doi: 10.1038/s41598-022-20218-3 (PMC9509316; doi:10.1038/s41598-022-20218-3)
Supplement: Supplementary file 1 — Supplementary Information. [file 41598_2022_20218_MOESM1_ESM.pdf]

Title: **Selective modulation of cortical population dynamics during neuroprosthetic skill learning**

Authors:

Ellen L. Zippi[1]*, Albert K. You[2]*, Karunesh Ganguly[3,4], and Jose M. Carmena[1,2+]

Affiliations:

[1] Helen Wills Neuroscience Institute, University of California Berkeley, Berkeley, California, USA, 94720

[2] Department of Electrical Engineering and Computer Sciences, University of California Berkeley, Berkeley, California, USA, 94720

[3] Neurology & Rehabilitation Service, San Francisco VA Medical Center, San Francisco, CA, USA, 94121

[4] Department of Neurology, University of California, San Francisco, CA, USA, 94143

* Authors contributed equally

[+] Corresponding author: jcarmena@berkeley.edu

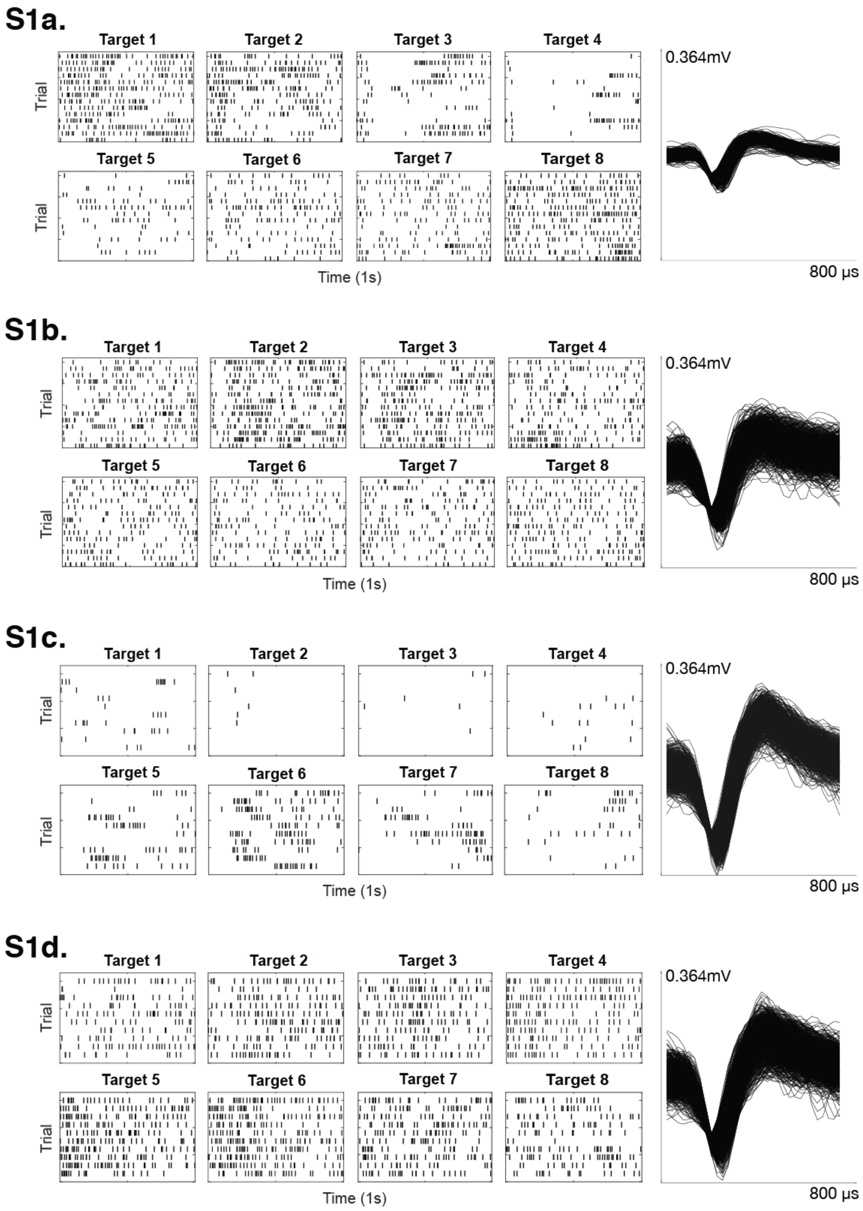

Figure S1. Spiking activity of representative units during BMI control. Raster plots separated by target (left) and 1000 waveforms (right). The height and width the waveform axes are 0.364 mV and 800 μs, respectively. (A) Spiking activity of a direct neuron from Monkey P. (B) Spiking activity of an indirect neuron from Monkey P. (C) Spiking activity of a direct neuron from Monkey R. (D) Spiking activity of an indirect neuron from Monkey P.

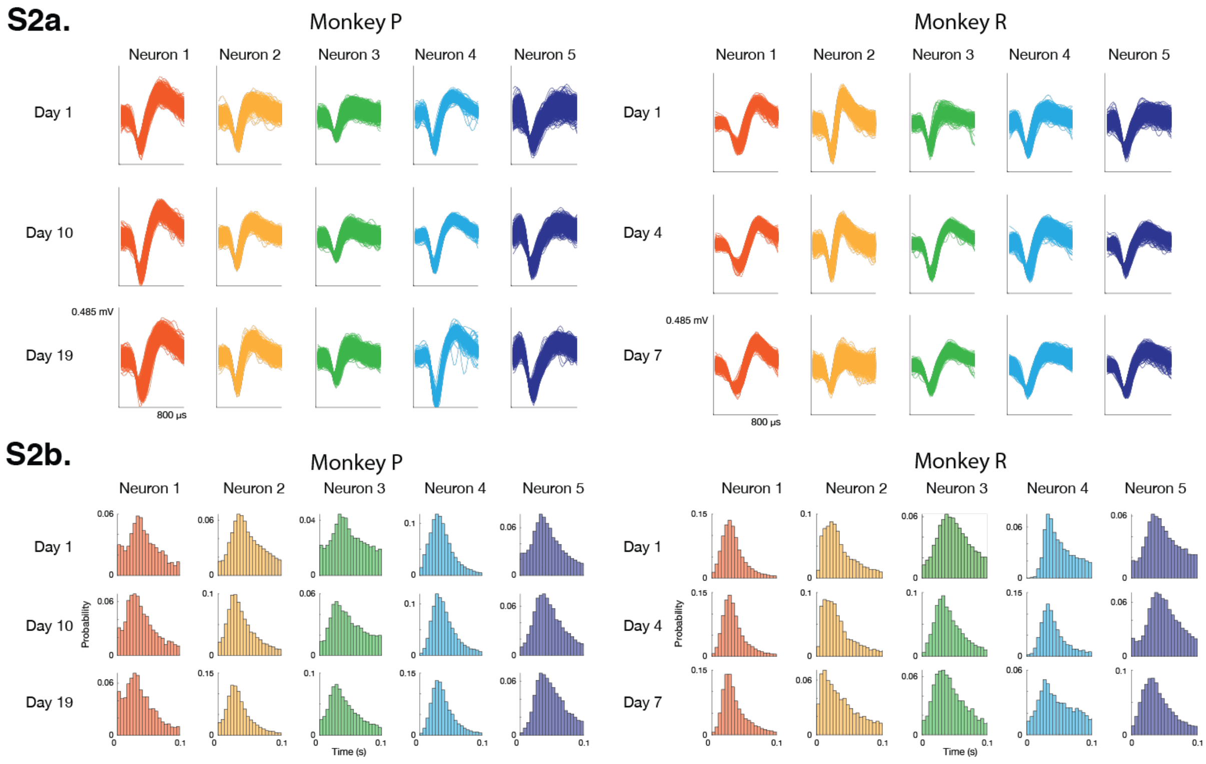

Figure S2. Stability of indirect neuron recordings. (A) Waveforms from five representative putative stable units as determined by the methods in Fraser & Schwartz, 2012 on the first day, middle day, and last day of recording for each animal. The height and width of each box are 0.485 mV and 800 μs, respectively. (B) Inter-spike interval (ISI) distributions are shown for the first day, middle day, and last day of recording for each animal for the same five representative stable units.

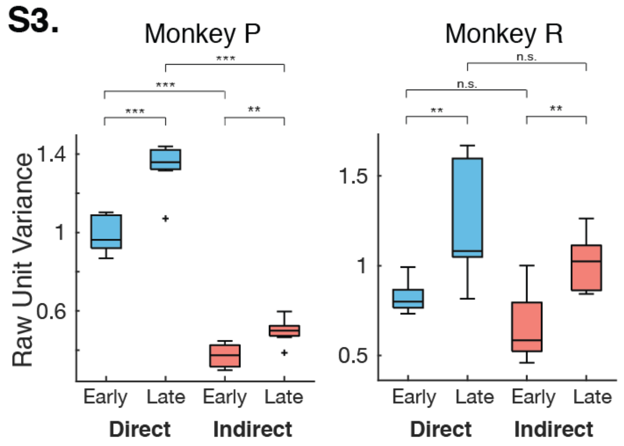

Figure S3. Raw neural variance in direct and indirect subpopulations. Variance was calculated for each neuron and then averaged across neurons. Both direct and indirect subpopulations increased neural variance from early to late learning (Unpaired t-test; Monkey P: direct p = 8.63e-5, indirect p = 0.002; Monkey R: direct p = 0.007, indirect p = 0.003). There is a difference in neural variance between the direct and indirect subpopulations in both early and late learning for Monkey P, but not for Monkey R (Unpaired t-test; Monkey P: early p = 5.15e-9, late p = 2.09e-8; Monkey R: early p = 0.060, late p = 0.113).

S4.

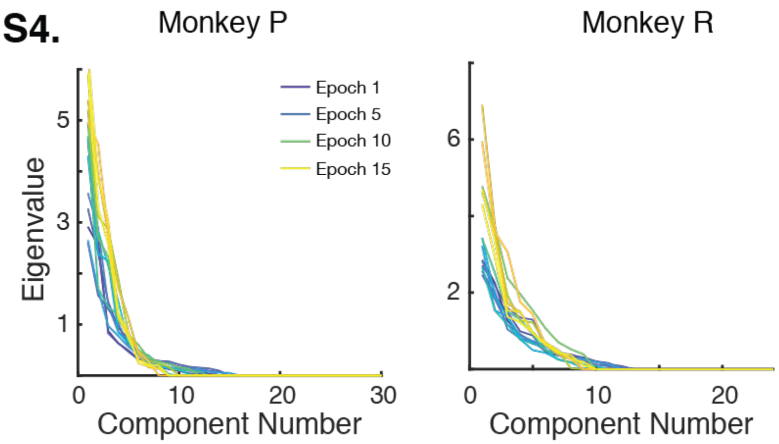

Figure S4. Scree plots for direct and indirect combined population. Factor analysis was performed on neural activity from the combined direct and indirect population for each training epoch individually. A scree plot is shown for each training epoch indicated by color.
